# Sternocleidomastoid size and upper trapezius muscle thickness in congenital torticollis patients: A retrospective observational study

**DOI:** 10.1097/MD.0000000000028466

**Published:** 2021-12-30

**Authors:** Dong Rak Kwon, Yoontae Kim

**Affiliations:** a Department of Rehabilitation Medicine, Catholic University of Daegu School of Medicine, 33 Duryugongwon-ro 17-gil, Nam-Gu, Daegu, South Korea; b Department of Physical Medicine and Rehabilitation, Soonchunhyang University Cheonan Hospital, Soonchunhyang University College of Medicine, Chungcheongnam-do, South Korea.

**Keywords:** accessory nerve, sternocleidomastoid muscle, torticollis, upper trapezius muscle

## Abstract

The purpose of this study was to investigate the upper trapezius muscle thickness (UTMT) in congenital muscular torticollis (CMT) patients and determine the correlation among sternocleidomastoid muscle thickness (SCMT), accessory nerve (AN) cross-sectional area (CSA), and UTMT in CMT.

This retrospective study consisted of 2 participant groups: Group 1 (SCM mass CMT, n = 20) and Group 2 (Postural CMT, n = 22). For both groups, B-mode ultrasound was performed by a physiatrist to measure the SCMT and UTMT and calculate the CSA of the AN. The correlation among SCMT, CSA of the AN, and UTMT in both groups was evaluated.

The between-group comparison revealed that Group 1 had significantly greater SCMT, UTMT, and CSA of the AN on the affected side than Group 2 (*P* < .05). The intragroup comparison between the affected and unaffected sides also revealed that, in Group 1, the SCMT, UTMT, and CSA of the AN were significantly higher on the affected side than on the unaffected side (*P* < .05), whereas no significant differences were observed in Group 2. In Group 1, a positive correlation (*r* = 0.55) was observed between the UTMT and CSA of the AN on the affected side, but not observed between the SCMT and CSA of the AN.

The findings of the study indicate that sternocleidomastoid muscle size may impact the thickness of the upper trapezius muscle via the accessory nerve in patients with congenital torticollis.

## 1 Introduction

Characterized by the shortening of the sternocleidomastoid muscle (SCM), congenital muscular torticollis (CMT) is a common congenital musculoskeletal disorder accompanied by a neck deformity.[^1^,^2^] Clinically, torticollis tends to have typical head tilting, restricted neck rotation, and/or a palpable mass.^[3]^

It has been reported that the prevalence rate of CMT was 0.3% to 2.0%.^[4]^ There have been several theories on CMT etiology. However, the exact cause of CMT remains unclear. Young children with persistent torticollis may have a higher risk for craniofacial anomalies or flat head syndrome.

A study suggests that only 20% among all torticollis cases do not spontaneously resolve without intervention.^[5]^ The first-line therapy for young children with CMT is physiotherapy, which comprises passive and active exercises as well as massage sessions. According to another study, CMT that persisted for ≥1 year since birth is unlikely to resolve by itself.^[6]^ Therefore, as shown in evidence-based guidelines for clinical practice,^[7]^ botulinum toxin injections are recommended for patients with head, neck, or trunk asymmetries that did not resolve after 4 to 6 weeks of comprehensive intervention, or for patients whose conservative treatment effects have entered into a stagnation phase after 6 months of treatment. This is to resolve the asymmetries and avoid any additional deformities or compensatory mechanisms.^[7]^ It has also been reported that patients with CMT may experience a sense of tightness in upper trapezius muscles. Nevertheless, rotation, extension, and lateral flexion may be possible in both SCM and upper trapezius muscles.[^8^,^9^,^10^] Similarly, a previous study showed that there was shortening and tightening of the SCM as well as muscular hypertrophy of upper trapezius and scalene muscles in CMT. Therefore, it is advisable that simultaneous injections are administered on neck muscles around the affected SCM, including trapezius muscles, scalene muscles, splenius capitis or splenius cervicis, and levator scapulae muscles, as well as on the SCM itself.[^8^,^9^,^11^,^12^]

Providing motor control of SCM and trapezius muscles, the spinal accessory nerve (AN) crosses the carotid triangle of the neck, then it either passes through (63%) or crosses under (37%) the SCM.^[13]^ The AN can be observed to pass posteriorly and inferiorly backward and downward from the SCM, above levator scapulae muscles, and toward trapezius muscles. This area, known as the lateral cervical triangle, is adjoined by the SCM, trapezius muscles, and the clavicle, ventrally, dorsally, and caudally, respectively.

Furthermore, muscle cramps may influence people with and without comorbidities. Cramps, particularly normal nocturnal cramps, including benign fasciculations, are likely to occur peripherally. It is presumed that the intramuscular regions of the axons are the sites that produce cramps,^[14]^ as these regions are generally suppressed by the nerve parts closer to the center of the body. Specifically, considering the local anatomy (i.e., origin, insertion, and AN pathway) of the SCM, the induction of muscle contractions including muscle cramps in the upper trapezius of CMT patients may be secondary to the physical compression of AN. Taking into account the importance of effective CMT treatment and in light of the aforementioned issues, we aim to explore the upper trapezius muscle thickness (UTMT) in CMT patients using ultrasound (US), and evaluate any correlation among SCM thickness (SCMT), AN cross-sectional area (CSA), and UTMT in CMT. To the best of our knowledge, this is the first study to report on such a diagnostic method using US in pediatric CMT patients.

## 2 Methods

### 2.1 Patients

In this study of retrospective chart review, we evaluated 77 patients who had visited the Department of Physical Medicine and Rehabilitation at Daegu Catholic University Medical Center for CMT between September 2017 and February 2018. All participants underwent neck US for an evaluation of CMT on the day of outpatient clinic visits as well as cervical spine radiography with anteroposterior and lateral views to rule out congenital bony abnormalities. In cases where diagnosis was uncertain, computerized tomography (neck) and magnetic resonance imaging (neck and/or brain, etc) imaging was performed for collecting additional information.

**Figure 1:**
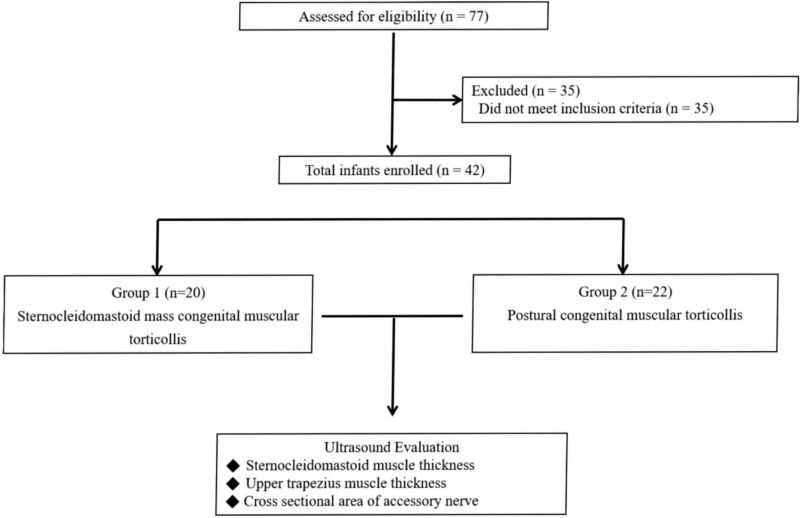
A flow diagram of the study.

Participants were selected for inclusion based on the criteria as follows: diagnosis of CMT confirmed by a physiatrist specializing in pediatric rehabilitation medicine, SCM mass CMT: patients with an SCM mass present with a fibrotic thickening of the SCM, passive range of motion limitations on clinical examination, and the difference of the thickness of the SCM muscle on both sides exceeded 2 mm on US of the neck,[^7^,^15^] postural CMT: patients have a postural preference without muscle tightness or limitation in passive range of motion on clinical examination, the difference of the thickness of the SCM muscle on both sides are <2 mm on US of the neck,[^7^,^15^] and no previous conventional medical treatment (e.g., physiotherapy) or evaluation of palpable mass was received prior to participation in this study. In contrast, the exclusion criteria were defined as follows: congenital cervical spine abnormalities (e.g., hemivertebra, atlanto-occipital dislocation, etc); cervical dystonia; neurogenic (syringomyelia, cerebral palsy, etc) and ocular torticollis; and soft tissue tumors, and inflammation or infections of the neck. Of the 77 evaluated patients, 42 were included in this study. Group 1 (SCM mass CMT n=20: male to female ratio, 15:5; mean age, 44.0 ± 10.9 days; and right-sided to left-sided mass ratio, 9:11) and Group 2 (postural CMT n = 22; male to female ratio, 14:8; mean age, 175.5 ± 39.4 days; and right-sided to left-sided mass ratio, 12:10) (Fig. 1).

All the study procedures were implemented after the study was approved by the Daegu Catholic University Research Ethics Committee (IRB no.: CR-18–039), and in accordance with the Declaration of Helsinki. Given the retrospective nature of this study, informed consent was not required.

### 2.2 Ultrasound examination

Using a commercially available US system, a physiatrist performed B-mode US scans with 5 to 13 MHz multi-frequency linear transducer (Antares; Siemens Healthcare, Erlangen, Germany). The physiatrist, who has had a 16-year experience in musculoskeletal US at the time of the investigation, reviewed all imaging findings. US was performed while the participants were sleeping, with assistance from their respective chaperones. For the examination, each participant was laid down on an examination couch for the operator to visualize the top of the head. The neck of each child was stretched maximally by placing a small supporting pillow under the neck while the head was rotated contralateral to the side examined. The procedure was suspended in cases of a tense or uncooperative infant.

**Figure 2:**
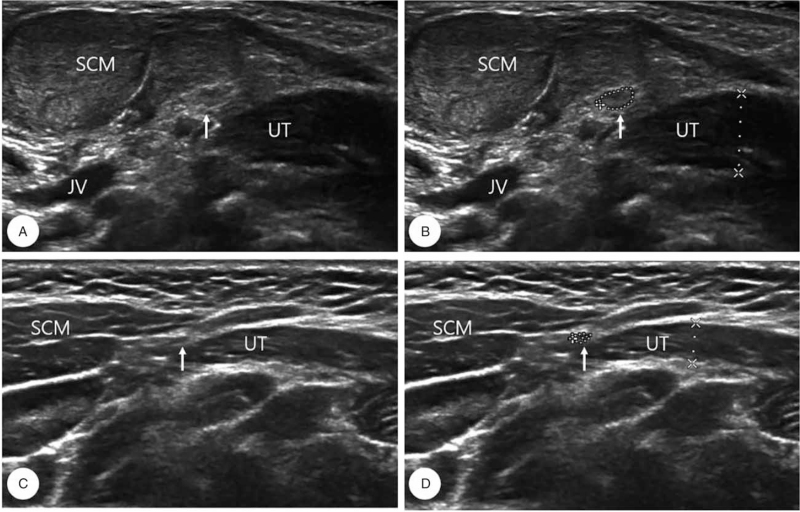
Illustrative transverse ultrasound images of the sternocleidomastoid muscle, accessory nerve, and upper trapezius in Group 1 (A, B) and Group 2 (C, D). (A, B) B-mode image showed fibrosis of sternocleidomastoid muscle with enlarged accessory nerve (arrow) in Group 1; (C, D) In Group 2, the cross-sectional area of accessory nerve and diameter of upper trapezius were measured. JV = jugular vein, SCM = sternocleidomastoid muscle, UT = upper trapezius muscle.

The bilateral SCMs of each participant were examined through the longitudinal (i.e., long axis of the muscle) and orthogonal (i.e., perpendicular to the long axis of the muscle) views. US images were recorded from the tumor site at the sternal or clavicular area of the SCM as well as the site of the mass in the lower, middle, or upper third of the muscle (Fig. 2A, C). The images showed that the AN was a small hypoechoic oval structure in the axial plane (Fig. 2B, D). The best view for AN identification was from the posterior triangle. After the identification of the upper trapezius muscle and SCM which were used as guiding structures, the transducer was moved superiorly, closer to the localization of the nerve. The paired AN was properly identified in all the study participants.

Muscle thickness was defined as the distance between the superficial and deep aponeuroses of the thickest part of the muscle in the axial view, measured using an electronic caliper (Fig. 2B, D). Based on the definition, SCMT and UTMT were measured. In addition, the CSA of the AN was evaluated (Fig. 2B, D). In both groups, SCMT, UTMT, and CSA of the AN were calculated. Moreover, the correlation between SCMT, CSA of the AN, and UTMT was evaluated in both groups. To improve the intra-rater reliability of the SCMT, CSA of the AN, and UTMT assessments, US scanning was conducted twice to take 2 illustrative US images in each scan.

### 2.3 Statistical analysis

The statistical analysis was performed using IBM SPSS ver. 19.0 (IBM Co., Armonk, NY). The level of significance was set at *P* < .05. The data were expressed as mean ± standard deviation or as frequency. Furthermore, the intra-rater reliability of repeated measurements of US parameters (i.e., SCMT, UTMT, and CSA of the AN) was expressed using the intraclass correlation coefficient. The independent *t* test was performed on continuous variables to evaluate the differences in US parameters between the 2 groups. Additionally, the correlation between numerical variables was calculated using Pearson correlation analysis.

## 3 Results

There was no significant difference in the sex ratios between the groups. However, the age at presentation was significantly higher in Group 2 than in Group 1. Using B-mode US, the palpable masses were detected in Group 1.

In Group 1, the SCMT, UTMT, and CSA of the AN on the affected side (11.83 ± 3.20, 5.10 ± 1.20 mm, and 1.26 ± 0.55 mm^2^, respectively) were significantly greater than that in Group 2 (6.74 ± 1.41, 3.12 ± 0.41 mm, and 0.47 ± 0.23 mm^2^, respectively) (*P* < .05, Table 1). For the intragroup comparison in Group 1, the SCMT, UTMT, and CSA of the AN on the affected side (11.83 ± 3.20, 5.10 ± 1.20 mm, and 1.26 ± 0.55 mm^2^, respectively) were significantly greater than those in the unaffected side (5.79 ± 0.73, 2.56 ± 0.44 mm, and 0.41 ± 0.15 mm^2^, respectively) (*P* < .05, Table 2). Meanwhile, no significant difference was observed in Group 2 (*P* > .05, Table 2). In Group 1, a positive correlation was found between the CSA of the AN and UTMT (*r* = 0.55, Table 3) on the affected side, but not between the CSA of the AN and SCMT. However, no correlation was found between SCMT, UTMT, and CSA of the AN on the affected side in Group 2.

**Table 1:** Comparative analysis of ultrasound values between sternocleidomastoid mass congenital muscular torticollis and postural congenital muscular torticollis.

Parameter	Group 1 (n = 20)	Group 2 (n = 22)	*P* value
SCM thickness, mm	11.83 ± 3.20	6.74 ± 1.41	.001^∗^
UT thickness, mm	5.10 ± 1.20	3.12 ± 0.41	.000^∗^
Accessory nerve CSA, mm^2^	1.26 ± 0.55	0.47 ± 0.23	.033^∗^

**Table 2:** Comparative analysis of ultrasound values in sternocleidomastoid mass congenital muscular torticollis and postural congenital muscular torticollis.

Parameter	Affected side	Unaffected side	*P* value
Group 1			
SCM thickness, mm	11.83 ± 3.20	5.79 ± 0.73	.000^∗^
UT thickness, mm	5.10 ± 1.20	2.56 ± 0.44	.000^∗^
Accessory nerve CSA, mm^2^	1.26 ± 0.55	0.41 ± 0.15	.000^∗^
Group 2			
SCM thickness, mm	6.74 ± 1.41	6.52 ± 1.33	.081
UT thickness, mm	3.12 ± 0.41	3.09 ± 0.50	.569
Accessory nerve CSA, mm^2^	0.47 ± 0.23	0.48 ± 0.21	.760

**Table 3:** Correlation of sternocleidomastoid muscle thickness, upper trapezius muscle thickness, and cross-sectional area of accessory nerve in patients with sternocleidomastoid mass congenital muscular torticollis.

Pearson correlation	SCM thickness	UT thickness	Accessory nerve CSA
SCM thickness, mm	1	0.397	0.131
UT thickness, mm	0.397	1	0.550^∗^
Accessory nerve CSA, mm^2^	0.131	0.550^∗^	1

The intraclass correlation coefficients for the repeated measurements of SCMT, UTMT, and CSA of the AN on the affected sides in Groups 1 and 2 were *r* = 0.905/0.910, *r* = 0.899/0.903, and *r* = 0.889/0.895, respectively.

## 4 Discussion

This retrospective study demonstrated that the UTMT of the affected SCM among young children with fibromatosis colli was significantly greater than in those with postural torticollis. Furthermore, the upper trapezius in the affected SCM was thicker than in the unaffected SCM. Among young children with fibromatosis colli, the affected SCM had a significantly greater CSA of the AN than the unaffected SCM. The results of this study may be explained by various factors.

First, it was assumed that, considering the anatomy, there was a synergistic effect on head rotation from the SCM and the upper trapezius muscle which were superficial in location and had the same main functions. For instance, in cervical dystonia patients, information would need to be collected to analyze abnormal rotational movements and identify muscles involved (e.g., sternocleidomastoid and upper trapezius muscles) for botulinum toxin injections.

Second, a recent study on surgical specimens of sternocleidomastoid tumors which were analyzed through electron microscopy and immunohistochemistry, demonstrated that myoblasts may be found in the proliferating interstitium of the tumor. The myoblasts may lead to the maturation and resolution of the tumor possibly through the production of unaffected myofibrils.^[16]^ The SCM shortening and/or the palpable mass may be correlated with repetitive small injuries, inflammation, and loss of muscle function. Repetitive small injuries, also known as muscle microtrauma, may lead to an elevation of calcium concentration, which is essential for transient changes in muscle excitation–contraction coupling. The sustained increase in calcium concentration may result in the activation of calcium-sensitive proteases and phospholipases,^[17]^ which would, in turn, damage the integrity of the cell membrane and sarcoplasmic reticulum due to possible changes in membrane permeability.[^16^,^18^] Another study suggested that a consistent rise in intracellular calcium ion concentration is related to the activation of non-lysosomal cysteine proteases such as calpain.^[17]^ Calpain separates various substrates of proteins, including cytoskeletal and myofibrillar proteins. Thus, calpain-mediated protein degradation may cause structural changes in the muscle.^[17]^ It is assumed that the decreased muscular function due to torticollis, contributes to morphological changes in the contractile machinery of the muscle.

Third, neurogenic causes that result in muscle fiber contraction, such as hyperirritability of the neuromuscular junction or muscle fiber, generate sensory feedback through the muscle spindle. Then, the sensory feedback turns into afferents of a spinal reflex which cause the activation of motor units. As aforementioned, both healthy and diseased people can be affected by muscle cramps. As electromyogram shows cramps as high-frequency discharges of motor unit potentials, passive muscle stretching may relieve muscle cramping. Meantime, stimulating the motor nerve at a point below an area of nerve block may induce muscle cramps.

Last, even when no abnormality was observed on electromyography, patients frequently complained of muscle discomfort. Functional nerve entrapment may occur at the site where the nerve passes through the muscle or connective tissues. We introduce the concept of a nerve entrapment point (NEP) based on clinical experience, and we know that NEP may cause various symptoms such as ischemic pain in the muscles below the compressed nerve due to sustained contraction. Seong^[19]^ defined NEP as the point where a muscle physically compresses a nerve, thus causing pain in the innervated region. In case of repeated microtrauma, the muscles tend to be tense and tender, and they entrap or compress the adjacent vasa nervorum, resulting in focal ischemia,^[19]^ which may, in turn, cause membrane hyperexcitability.[^20^,^21^] Nerve hyperexcitation may generate a signal of abnormal excitation and result in various types of ischemic pain. Based on our experience and on previously published reports, it is recommended that CMT patients with no response to a home-based stretching program or physical therapy should be considered candidates for intensive physical therapy with microcurrent therapy or botulinum toxin injections into the affected cervical muscles. The appropriate intervention should be done to reduce pain and muscle spasm, prevent muscle fibrosis and contracture, and improve long-term outcomes. A previous study^[22]^ introduced the use of microcurrents in physical therapy. This randomized controlled trial compared therapeutic exercise and US with and without additional microcurrent therapy. The results showed that a group with microcurrent therapy had a shorter duration of treatment, a higher improvement of neck rotation by passive ROM, and a lesser thickness, CSA, and red pixel intensity of the involved SCM than a group without microcurrent therapy.^[22]^ Another study^[10]^ demonstrated that botulinum toxin treatment increased the degree of ROM and head tilt and improved the changes in SCMT and SCM length. The efficacy rate of the combined management of physical therapy and botulinum toxin injections was calculated to be 84%, which suggests that a local botulinum toxin injection would have positive impact on CMT prognosis.^[10]^

In this study, the difference in age at presentation between fibromatosis colli and postural torticollis patients was found to be significant. This is consistent with a previous study by Han et al^[23]^ in which clinical characteristics of infantile CMT patients with and without an SCM lesion detected using US were compared, and those with an SCM lesion tended to visit the clinic at a younger age. In these patients, there were greater restrictions in cervical rotation and lateral flexion, a higher rate of breech presentations, a lower rate of cesarean deliveries, and a lower prevalence of plagiocephaly than in those without an SCM lesion.^[23]^

Having been recently used to identify fibrotic lesions within the SCM, US is believed to have a higher sensitivity in differentiating CMT from other cervical diseases.^[24]^ In muscular torticollis, early diagnosis and appropriate therapeutic interventions are critical for better management outcomes. However, to the best of our knowledge, there have been no studies which evaluated the same parameters in CMT patients with SCM masses. In this study, we evaluated the CSA of the AN and UTMT in CMT patients with an SCM mass using real-time US. US is a very useful diagnostic technique that allows the assessment of several parameters (e.g., CSA of the AN and UTMT) in an SCM mass to determine appropriate management options. Furthermore, it may be used to predict prognoses and monitor long-term progresses after treatment onset.

The findings of the study show that the thickness and CSA measurements on the affected side have excellent reliability. The result is consistent with previous studies, which showed good reliability of in vivo US assessment of local muscle thickness.[^25^,^26^,^27^,^28^,^29^]

Several limitations should be addressed. First, unfortunately, the imaging was conducted by only one physiatrist due to a lack of cooperation from the infant participants. For this reason, an interobserver reproducibility evaluation was unavailable. However, the intra-rater reliability of repeated measurements of US parameters ranged from 0.895 to 0.910, which indicates “good” to “excellent” reliability.^[30]^ Second, given the retrospective nature of the study, there were some difficulties during the collection of comprehensive information from the medical chart. Third, the number of cases included in this study was relatively small. Although 42 infants ultimately included in the analysis showed statistical significance, a larger study will be needed to validate the results. Last, the findings of the study provided comparative data on the diagnostic and prognostic strategies for CMT, yet, a definite conclusion regarding the diagnostic efficacy of US cannot be drawn due to the limited number of study participants. Therefore, further investigation should be conducted.

## 5 Conclusion

In this study, we demonstrated that sternocleidomastoid muscle size may affect the thickness of the upper trapezius muscles through the accessory nerve in patients with congenital torticollis.

## Author contributions

Dong Rak Kwon contributed to the study of conception and design. Dong Rak Kwon and Yuntae Kim contributed to the interpretations of the study results, writing, and editing of this manuscript.

**Conceptualization:** Dong Rak Kwon, Yoontae Kim.

**Data curation:** Dong Rak Kwon, Yoontae Kim.

**Formal analysis:** Dong Rak Kwon.

**Funding acquisition:** Dong Rak Kwon.

**Investigation:** Dong Rak Kwon.

**Methodology:** Dong Rak Kwon.

**Project administration:** Dong Rak Kwon.

**Supervision:** Dong Rak Kwon.

**Validation:** Dong Rak Kwon.

**Visualization:** Dong Rak Kwon.

**Writing – original draft:** Dong Rak Kwon, Yoontae Kim.

**Writing – review & editing:** Dong Rak Kwon, Yoontae Kim.
